# Progressive Changes in Brain Morphology in People With Idiopathic Generalized Epilepsy

**DOI:** 10.1212/WNL.0000000000214647

**Published:** 2026-01-28

**Authors:** Fenglai Xiao, Yingying Zhang, Britta Wandschneider, Lawrence P. Binding, Davide Giampiccolo, Marine Fleury, Luisa Delazer, Bernardo Crespo Pimentel, Isha Puntambekar, Kazuki Fukuma, Lorenzo Caciagli, Josemir W. Sander, John S. Duncan, Dong Zhou, Matthias J. Koepp

**Affiliations:** 1 Department of Clinical & Experimental Epilepsy, UCL Queen Square Institute of Neurology, London, United Kingdom;; 2 Chalfont Centre for Epilepsy, Chalfont St Peter, United Kingdom;; 3 Department of Neurology, West China Hospital, Sichuan University, Chengdu, China;; 4 Centre for Medical Image Computing, UCL Departments of Computer Science, Medical Physics, and Biomedical Engineering, London, United Kingdom;; 5 Victor Horsley Department of Neurosurgery, National Hospital for Neurology and Neurosurgery, London, United Kingdom;; 6 Department of Neurology, Medical University of Innsbruck, Austria;; 7 Department of Neurology, Neurointensive Care and Neurorehabilitation, Christian Doppler University Hospital, Paracelsus Medical University, Centre for Cognitive Neuroscience Salzburg, Austria;; 8 Department of Neurology, Inselspital, Sleep-Wake-Epilepsy-Center, Bern University Hospital, University of Bern, Switzerland; and; 9 Centre for Global Epilepsy, Wolfson College, Oxford, United Kingdom.

## Abstract

**Background and Objectives:**

Idiopathic generalized epilepsy (IGE) is typically responsive to treatment, yet some people remain poorly controlled despite multiple antiseizure medication trials. Whether this subgroup shows progressive structural brain changes remains uncertain. We aimed to investigate whether people with poorly controlled chronic IGE exhibit progressive morphological brain alterations and how these differ from those in early-stage IGE.

**Methods:**

This longitudinal case-control neuroimaging study included 2 cohorts: an early-stage IGE group from West China Hospital (Chengdu, China) and a chronic IGE group from the National Hospital for Neurology and Neurosurgery (London, United Kingdom). Participants had a history of generalized seizures and no focal pathology. Matched healthy controls were drawn from 3 public imaging data sets. Participants underwent 2 high-resolution T1-weighted MRI scans at least 12 months apart. Cortical thickness and hippocampal and subcortical volumes were measured on paired scans. Longitudinal changes were assessed using linear mixed-effects models, correcting for age and interscan interval. Structural covariance analysis was conducted to examine inter-regional relationships over time, focusing on thalamo-cortical coupling.

**Results:**

Forty-two people with early-stage IGE and 67 with chronic IGE were recruited from 2 separate epilepsy centers, and 109 matched controls were included. The early-stage IGE group showed progressive atrophy limited to the left putamen. Chronic IGE was associated with widespread cortical thinning, primarily in frontal and temporal regions, and thickening in posterior and occipital areas. Subcortical atrophy involves the putamen, thalamus, and pallidum. People with ongoing generalized tonic-clonic seizures showed thickening in the precentral gyrus and additional thinning in the frontal cortex and precuneus. Photosensitive IGE was associated with thickening in the lingual gyrus and occipital cortex. Valproate use was associated with attenuated structural changes in motor, visual, and subcortical regions. Increased structural covariance was observed between the left thalamus and left lingual gyrus in chronic IGE.

**Discussion:**

Chronic IGE, particularly with persistent generalized tonic-clonic seizures or photosensitivity, is associated with more extensive progressive brain changes compared with matched controls. Valproate may have a protective association with these structural changes, although further validation is needed. Longitudinal MRI may help monitor disease progression and treatment effects in poorly controlled IGE.

## Introduction

Idiopathic generalized epilepsy (IGE) constitutes approximately one-fifth of all epilepsies, encompassing syndromes such as childhood absence epilepsy, juvenile absence epilepsy (JAE), juvenile myoclonic epilepsy (JME), and epilepsy with generalized tonic-clonic seizures (GTCSs) alone (GTCA).^1^ IGEs typically present in childhood or adolescence and often share overlapping clinical features, including normal neurologic development, good prognosis for seizure control, and 2.5–6 Hz generalized spike-wave or polyspike-wave discharges on EEG, often activated by hyperventilation or photic stimulation.^1^

IGEs are usually responsive to antiseizure medications (ASMs), with over 70% being seizure-free.^1,2^ However, relapse after ASM withdrawal is common,^1,2^ and many adults experience GTCSs, which are associated with increased risks of injury and sudden unexpected death in epilepsy.^3^ Treatment decisions are particularly challenging for women of childbearing potential because sodium valproate (VPA)—the most effective drug for JME and GTCSs—carries significant teratogenic risks.^3^

Although often treatment-responsive, a subset develops a chronic, drug-resistant form of IGE that remains underascertained.^3^ Structural MRI, by measuring cortical thickness and subcortical volumes, provides a quantitative biomarker to track disease progression.^4^ We previously identified distinct patterns of atrophy in IGE using a machine-learning model, with progressive basal ganglia atrophy linked to frequent GTCSs and poor outcomes.^5^ The divergence in structural trajectories between drug-responsive and drug-resistant IGE remains unclear.

Longitudinal neuroimaging is superior to cross-sectional studies for assessing disease progression.^6^ A longitudinal structural assessment of new-onset JME within 2 years of diagnosis found abnormal neurodevelopment linked to alterations in fronto-parietal-temporal association cortices.^7^ Our recent longitudinal study in adults with focal epilepsies showed faster rates of annualized cortical thinning than in controls.^8^ These observations have focused on the cortex. However, evidence from magnetic stimulation^9^ and neuroimaging^10^ increasingly underscores the critical role of subcortical structure, especially the thalamus and basal ganglia, in IGE pathogenesis.

In this study, we examined longitudinal brain changes in a cohort with early-stage IGE and a separate cohort with chronic IGE, compared with matched healthy controls (HCs). We aimed to (1) assess whether chronic IGE shows more pronounced progressive atrophy than early-stage IGE; (2) investigate associations between structural changes and key clinical features (GTCSs, photosensitivity, treatment response); (3) differentiate structural trajectories between drug-responsive and drug-resistant IGE; and (4) explore altered thalamo-cortical structural covariance in chronic IGE.

## Methods

### Participants

We studied 2 distinct IGE cohorts. (1) Early-stage IGE cohort: We recruited individuals with a confirmed International League Against Epilepsy diagnosis of IGE, a disease duration of <5 years, and seizure freedom for ≥1 year. All underwent two 3T MRI scans ≥12 months apart (2013–2024) at West China Hospital, Chengdu, China. Diagnosis was based on a typical adolescent onset of myoclonic, absence, or GTCSs; interictal generalized spike-wave discharges on EEG; and the absence of structural abnormalities on MRI.^1^ (2) Chronic IGE cohort: Participants with IGE duration >10 years were identified from a clinical registry at UCL Queen Square Institute of Neurology, London, United Kingdom. Diagnosis was confirmed through medical records and historical VEEG, with photic stimulation showing generalized discharges. Clinical data were collected from medical notes nearest the scan dates. We focused on active GTCSs in the year before the second scan as a reliable measure of seizure activity.^11^ They underwent 2 clinical 3T MRI scans ≥12 months apart (2004–2022) with no reported abnormalities. Scanner details are given in eMethods 1.

We used anonymized MRI data from HCs without neurologic or psychiatric disorders in 3 public studies (eMethods 2, eTable 1). To match our early-stage IGE cohort with age (17–25) and ethnicity, we selected 42 controls from the Southwest University Longitudinal Imaging Multimodal (SLIM) study from Chongqing, China.^12^ For age-matching (18–70 years) with our UK cohort, we incorporated data from the Social Schizophrenia Initiative in Neurobiology of the Schizophrenia(s) (SPINS)^13^ study and the Parkinson's Progression Markers Initiative (PPMI)^14^ study. Controls were matched to the epilepsy cohorts based on age, sex, and the availability of 2 high-resolution T1-weighted scans spaced ≥12 months apart.

### Standard Protocol Approvals, Registrations, and Participant Consents

The study was approved by the Joint Research Ethics Committee of UCL and University College London Hospital (20/LO/0149), and no informed consent was required for analyzing previously acquired clinical data. The West China Hospital Clinical Trials and Biomedical Ethics Committee approved recruitment for the early-stage cohort (20220909), and participants provided written informed consent.

### MRI Preprocessing

Cortical thickness was estimated using the Computational Anatomy Toolbox (CAT12) with an inverse-consistent longitudinal surface registration approach to prevent an asymmetry bias from arising when data from multiple time points are analyzed.^15^ We used the Hipposeg algorithm,^16^ an open-source, multiatlas-based, previously validated hippocampal segmentation algorithm, for accurate measurement of hippocampal volume.^17^ Hipposeg delineates the hippocampus with variability no greater than seen between expert human raters and is robust to hippocampal morphological alterations, including atrophy. It was built using 876 3T and 202 1.5T scans of people with epilepsy. It has continuously improved and demonstrated superior delineation of diseased hippocampi compared with other automated segmentation methods.^18^ The subcortical structures and total intracranial volume were measured using the Geodesic Information Flows algorithm,^19,20^ validated within the neurodegenerative diseases research.^3,4^ Both algorithms are accessible on NiftyWeb.^21^ MRI data were adjusted for scanner-related batch effects using the validated ComBat Tool for Harmonization of Multi-Site Imaging Data (eMethods 3).^20^ To verify that site differences did not confound results, we applied ComBat harmonization using the site as the batch variable without including biological covariates. ComBat harmonization did not materially alter the data (eMethods 3, eTables 2 and 3): all within-site paired comparisons of cortical thickness and subcortical volumes (left/right hippocampus, thalamus, caudate, putamen, pallidum) were nonsignificant before vs after ComBat harmonization. Diagnostics (eMethods 3, eTable 4) showed modest reductions in site terms and no systematic variance shifts (Brown-Forsythe). Longitudinal time effects were stable with and without ComBat harmonization and robust to leave-one-site-out checks (eMethods 3, eFigures 1 and 2).

### Statistical Analysis

Demographic and clinical data were compared using analysis of variance, Kruskal-Wallis tests, and χ^2^ tests (SPSS 29 and R 4.2.1).

Vertex-wise cortical thickness measurements were analyzed using CAT12 and integrated into a flexible factorial design for longitudinal data across groups, examining covariate-by-group interactions. The dependent variable was cortical thickness at each vertex, entered at both time points. The model included a within-participant factor of time (baseline/follow-up) and continuous covariates for age, sex, and interscan interval. This allowed us to assess longitudinal cortical change while accounting for interindividual variability in scan timing. For region-wise analyses of hippocampal and subcortical volumes, we applied linear mixed-effects models in SPSS, with the dependent variable being the volume at each time point. Group, time point (baseline/follow-up), and their interaction were modeled as fixed effects, along with age, sex, and interscan interval. A participant was modeled as a random intercept to account for repeated measures. “Random participant effects” refers specifically to participant-wise random intercepts only. These models assessed changes over time differing between groups and the influence of clinical variables on cortical thickness or subcortical volumes, correcting for baseline demographic variations and interscan intervals.^22^ For subgroup analysis, we divided the chronic cohort into 2 groups based on whether they (1) had active GTCSs during the year before the second scan, (2) were photosensitive, (3) were taking VPA between the 2 scans.^23^ The early-stage cohort was stratified by GTCS history. Propensity score matching (R) controlled for age, sex, onset age, scan interval, ASM, and syndrome (eMethods 4).

Cortical thickness contrasts were computed using threshold-free cluster enhancement (10,000 permutations, *p* < 0.05, family-wise error [FWE] corrected; eMethods 5). Cohen *d* was estimated from the magnitude of *t*-statistics surviving FWE correction. Subcortical and hippocampal volumes were tested at *p* < 0.05 (Bonferroni corrected). All models accounted for random participant and fixed effects of age, sex, and group, adjusting for age and interscan interval differences.

Morphometric correlation analysis^24^ explored subcortical-cortical structural covariance using CAT12. We assessed covariance separately at each time point to examine evolving network architecture over time. Although no specific biological event was hypothesized between scans, this approach enables the investigation of dynamic alterations in thalamo-cortical covariance during chronic progression. At each scanning time point, we correlated the volumes of subcortical regions in native space with cortical thickness at each vertex. Significant correlations were interpreted as connections, and statistical significance was set at *p* < 0.05, FWE corrected. Then, we fitted linear models at each vertex, including a repeated-scans main-effect term, a subcortical-volume main-effect term, and a time×subcortical volume interaction-effect term. We assessed differences in subcortical-cortical correlations between the repeated scans by testing the significance of the interaction term at each vertex.^24,25^

### Data Availability

Anonymized data supporting the main findings are available from the corresponding authors on reasonable request.

## Results

We included 42 participants with early-stage IGE and 42 age-matched and sex-matched HCs from the SLIM study. Comparison between those with early-stage IGE (n = 42, 52% female, mean age 17.93 ± 3.33 years) and HCs (n = 42, 50% female, mean age 18.83 ± 1.82 years) showed no significant differences in sex (χ^2^ = 0.048, *p* = 1.00) or age (*F* = 1.141, *p* = 0.294). People in the early stage had a mean onset age of 13.74 ± 2.95 years, had a disease duration of 4.21 ± 3.32 years, and were seizure free for ≥1 year before follow-up; 34 of 42 had a history of GTCSs, and 36% used VPA treatment. We included 67 participants with chronic IGE and 67 age-matched and sex-matched HCs from PPMI (n = 64) and SPINS (n = 3) studies. The chronic IGE cohort (n = 67, 48% female, mean age 34.54 ± 12.63 years) showed no significant differences from HCs of PPMI/SPINS (n = 67, 51% female, mean age 35.44 ± 12.62 years) in sex distribution (χ^2^ = 0.123, *p* = 0.86) or baseline age (*F* = 0.413, *p* = 0.68). Participants with chronic IGE had a mean onset age of 13.79 ± 7.0 years and a mean disease duration of 19.48 ± 11.31 years, with all 67 having a history of GTCS. At the time of the second scan, 54 of 67 had active GTCSs (16 weekly/22 monthly/15 >twice/year) while 4 of 67 were seizure free. IGE syndromes included GTCA (43) and JAE/JME (24), with 38% on VPA. Photosensitivity was present on EEG in 42 of 67. ASM use ranged from 1 to 4 (median = 2). Active GTCSs did not significantly correlate with VPA use (χ^2^ = 1.644, *p* = 0.20) (Table 1). The early-stage cohort had a similar interscan interval to matched HCs (mean [SD] 2.7 [2.2] vs 2.7 [2.0], *p* = 0.83), whereas the interval was longer in the chronic cohort (6.3 [4.1] years vs 3.1 [2.0], *p* < 0.001); this was corrected in later analyses. Detailed cortical and subcortical results are described in the supplemental material (eTables 10–20). There was a notable imbalance between participants in the chronic cohort with GTCSs (n = 54) and those without (n = 13). To optimize statistical power while maintaining reasonable comparability, we applied 2:1 matching for this subgroup analysis. The propensity matching score for subgroup analysis is given in eTables 5–9.

**Table 1:** Clinical and Demographic Information of Patients With IGE and HCs

Characteristic	Early-stage IGE	HCs (SLIM)	Statistics	*p* Value
Sex, female/male, n (percentage of female)	22/20 (52)	21/21 (50)	0.048^a^	1.00
Age at baseline scan, y, mean (SD)	17.93 (3.33)	18.55 (1.82)	1.141	0.294
Interval between 2 scans, y, mean (SD)	2.7 (2.2)	2.7 (2.0)	0.45	0.830
Age at onset, y, mean (SD)	13.74 (2.95)	N/A	N/A	N/A
Duration, y, mean (SD)	4.21 (3.32)	N/A	N/A	N/A
Diagnosis: GTCA/JAE/JME, n	8/6/28	N/A	N/A	N/A
Free of GTCSs: yes/no, n	42/0	N/A	N/A	N/A
History of GTCSs: yes/no, n	34/8	N/A	N/A	N/A
No. of ASMs, n, median (range)	1 (1–2)	N/A	N/A	N/A
VPA treatment, n (percentage)	15 (36)	N/A	N/A	N/A

Characteristic	Chronic IGE	HC (PPMI/SPINS)	Statistics	*p* Value
Sex, female/male, n (percentage of female)	29/38 (48)	27/40 (51)	0.123^a^	0.86
Age at baseline scan, y, mean (SD)	34.54 (12.63)	35.44 (12.62)	0.413	0.68
Interval between 2 scans, y, mean (SD)	6.3 (4.1)	3.1 (2.0)	30.566	<0.001
Age at onset, y, mean (SD)	13.79 (7.0)	N/A	N/A	N/A
Duration, y, mean (SD)	19.48 (11.31)	N/A	N/A	N/A
Diagnosis: GTCA/JAE or JME, n	43/24	N/A	N/A	N/A
History of GTCSs, yes/no, n	67/0	N/A	N/A	N/A
Active GTCSs before the second scan, yes/no, n	54/13	N/A	N/A	N/A
GTCS frequency before the second scan (n = 54), weekly/monthly/at least twice a year	16/22/15			
Seizure free before the second scan, yes/no, no	4/63	N/A	N/A	N/A
Photosensitivity at video EEG +/−	42/25	N/A	N/A	N/A
No. of ASMs, n (range)	2 (1–4)	N/A	N/A	N/A
VPA, n (percentage)	24 (38)	N/A	N/A	N/A
VPA frequency in those with active GTCS and those without	15/54: 6/13	N/A	1.644^a^	χ^2^: 0.20

Abbreviations: ASM = antiseizure medication; GTCA = GTCS-alone; GTCS = generalized tonic-clonic seizure; HCs = healthy controls; JAE = juvenile absence epilepsy; JME = juvenile myoclonic epilepsy; N/A = not available; PPMI = Parkinson's Progression Markers Initiative; SLIM = Southwest University Longitudinal Imaging Multimodal; SPINS = Social Processes Initiative in Neurobiology of the Schizophrenia(s); VPA = valproate.

### Associations of Disease Stage: Early and Chronic IGE

**Figure 1:**
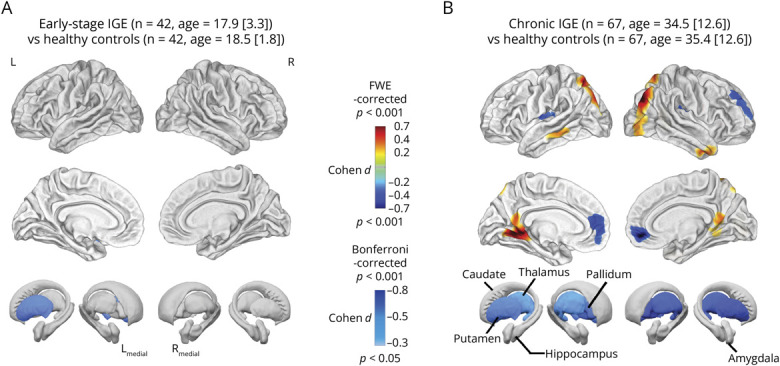
Association of Disease Stages: Early and Chronic IGE (A) In the early-stage IGE cohort compared with matched healthy controls, no significant changes in cortical thickness were observed. However, progressive atrophy of the left putamen was detected between the 2 scans (*p* < 0.05, Bonferroni-corrected). (B) In the chronic IGE cohort compared with matched controls, vertex-wise analysis revealed progressive cortical thinning in the superior and frontal gyri and cortical thickening in the visual cortices and bilateral middle temporal gyri at follow-up (*p* < 0.05, FWE-corrected). Progressive subcortical atrophy was also observed in the bilateral pallidum, putamen, and thalamus (*p* < 0.05, Bonferroni-corrected). Age and interscan intervals were corrected in the chronic IGE vs control analysis. FWE = family-wise error; IGE = idiopathic generalized epilepsy.

In the early-onset cohort, compared with matched controls (Figure 1A, eTable 10), we observed progressive atrophy in the left putamen (*p* = 0.045_Bonferroni_, *d* = 0.50) but no cortical thickness changes.

In the chronic cohort, compared with matched controls (Figure 1B, eTables 11 and 12), cortical thinning was observed in the right superior/middle frontal gyri (2,141 vertices, *p* < 0.001_FWE_, *d* = −0.58), left transverse temporal gyrus (1,573 vertices, *p* < 0.001_FWE_, *d* = −0.42), right anterior cingulate cortex (1,301 vertices, *p* = 0.011_FWE_, *d* = −0.43), and right rolandic operculum (1,282 vertices, *p* = 0.014_FWE_, *d* = −0.41). Cortical thickening was seen in the right middle occipital gyrus and calcarine sulcus (6,260 vertices, *p* < 0.001_FWE_, *d* = 0.65), left middle occipital gyrus (1,861 vertices, *p* = 0.007_FWE_, *d* = 0.45), lingual gyri (3,515 vertices, *p* < 0.001_FWE_, *d* = 0.60), bilateral superior parietal lobes (left: 1,642 vertices, *p* = 0.003_FWE_, *d* = 0.48; right: 1,332 vertices, *p* = 0.015_FWE_, *d* = 0.42), and bilateral temporal gyri (left: 965 vertices, *p* = 0.041_FWE_, *d* = 0.36; right: 837 vertices, *p* = 0.013_FWE_, *d* = 0.40). Subcortically, progressive atrophy was observed bilaterally in the putamen (left: *p* = 0.012, *d* = −0.68 right: *p* = 0.001_Bonferroni_, *d* = −0.70), thalamus (left: *p* = 0.039_Bonferroni_, *d* = −0.50, right: *p* = 0.003_Bonferroni_, *d* = −0.65), and pallidum (left: *p* = 0.003_Bonferroni_, *d* = −0.60, right: *p* = 0.001_Bonferroni_, *d* = −0.70).

### Association of GTCSs

**Figure 2:**
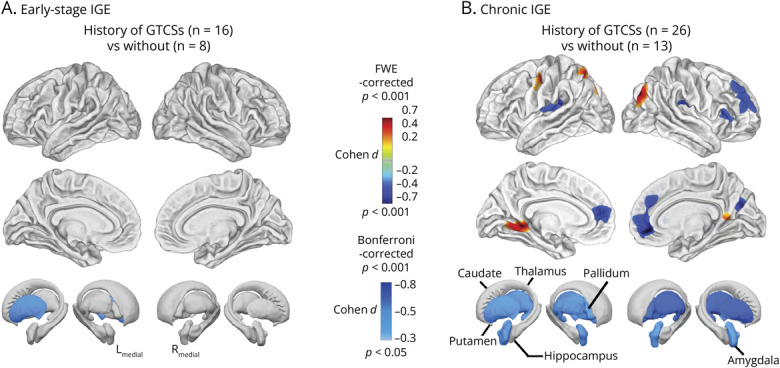
Association With GTCS (A) In the early-stage IGE cohort, participants with a history of GTCSs showed progressive atrophy of the left putamen compared with those without GTCSs (*p* < 0.05, Bonferroni-corrected). (B) In the chronic IGE cohort, participants with ongoing GTCSs exhibited progressive cortical thinning in the superior and frontal gyri and cortical thickening in the visual cortices and left precentral gyrus (*p* < 0.05, FWE-corrected). Subcortical atrophy was observed in the bilateral amygdala, pallidum, putamen, and thalamus (*p* < 0.05, Bonferroni-corrected). FWE = family-wise error; GTCSs = generalized tonic-clonic seizures; IGE = idiopathic generalized epilepsy.

In the early-stage cohort, participants were seizure free during the year preceding the second scan. Sixteen with a history of GTCSs were matched to 8 without, showing progressive left putamen atrophy (*p* = 0.022_Bonferroni_, *d* = −0.55) relative to those without (Figure 2A, eTable 13).

In the chronic cohort, those with active GTCSs showed cortical thinning relative to those without in the left superior temporal (2,462 vertices, *p* < 0.001_FWE_, *d* = −0.59), right rolandic operculum (2,496 vertices, *p* < 0.001_FWE_, *d* = −0.60), and bilateral inferior/middle frontal gyri (left: 2,000 vertices *p* < 0.001_FWE_, *d* = −0.56; right: 5,801 vertices, *p* < 0.001_FWE_, *d* = −0.69). Cortical thickening was seen in the right middle occipital gyrus (1,408 vertices, *p* = 0.006_FWE_, *d* = 0.43), left lingual gyrus (1,729 vertices, *p* = 0.004_FWE_, *d* = 0.46), left superior parietal lobe (1,233 vertices, *p* = 0.035_FWE_, *d* = 0.41), and left precentral gyrus (712 vertices, *p* = 0.027_FWE_, *d* = 0.39). Subcortically, progressive atrophy was observed bilaterally in the thalamus (left: *p* = 0.024_Bonferroni_, *d* = −0.55, right: *p* = 0.003_Bonferroni_, *d* = −0.70), putamen (left: *p* = 0.025_Bonferroni_, *d* = −0.55; right: *p* = 0.002_Bonferroni_, *d* = −0.73), pallidum (left: *p* = 0.019_Bonferroni_, *d* = −0.65; right: *p* = 0.005_Bonferroni_, *d* = −0.68), and left amygdala (*p* = 0.023_Bonferroni_, *d* = −0.60) (Figure 2B, eTables 14 and 15).

### Association of Photosensitivity

**Figure 3:**
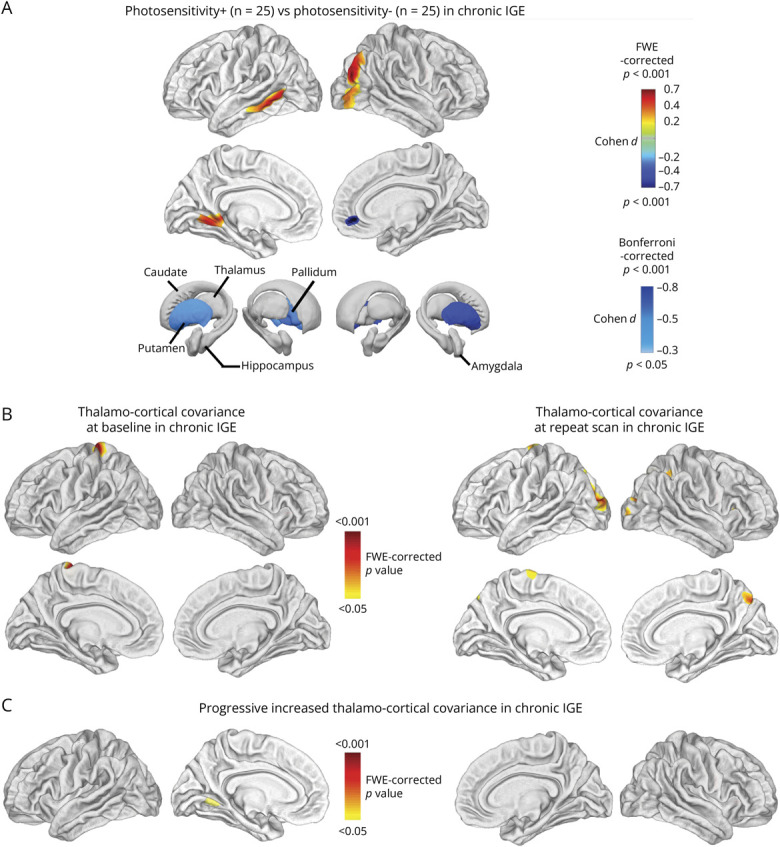
Association With Photosensitivity and Thalamo-Cortical Structural Covariance (A) In the chronic IGE cohort, participants with photosensitivity showed cortical thickening in the visual cortices (*p* < 0.05, FWE-corrected), along with atrophy in the left pallidum and bilateral putamen (*p* < 0.05, Bonferroni-corrected). (B) Structural covariance analysis showed increased covariance between the left thalamus and the left postcentral gyrus at both baseline and follow-up. At follow-up, additional covariance emerged between the left thalamus and occipital gyri associated with visual processing (*p* < 0.05, FWE-corrected). (C) Interscan difference maps showed increased covariance between the left thalamus and left lingual gyrus (*p* < 0.05, FWE-corrected). FWE = family-wise error; IGE = idiopathic generalized epilepsy.

Twenty-five participants with photosensitivity were matched to 25 without. In the chronic cohort, participants with photosensitivity showed cortical thickening in the right middle occipital gyrus (3,159 vertices, *p* = 0.004_FWE_, *d* = 0.65), left lingual gyrus (3,515 vertices, *p* = 0.025_FWE_, *d* = 0.55), and left middle temporal gyrus (1,861 vertices, *p* = 0.049_FWE_, *d* = 0.42). Cortical thinning was noted in the right orbitofrontal cortex (369 vertices, *p* = 0.004_FWE_, *d* = −0.40) and subcortical progressive atrophy in the bilateral putamen (left: *p* = 0.034_Bonferroni_, *d* = −0.55; right: *p* < 0.001_Bonferroni_, *d* = −0.82) and left pallidum (*p* = 0.025_Bonferroni_, *d* = −0.58) (Figure 3A, eTables 16 and 17).

### Association of Valproate

**Figure 4:**
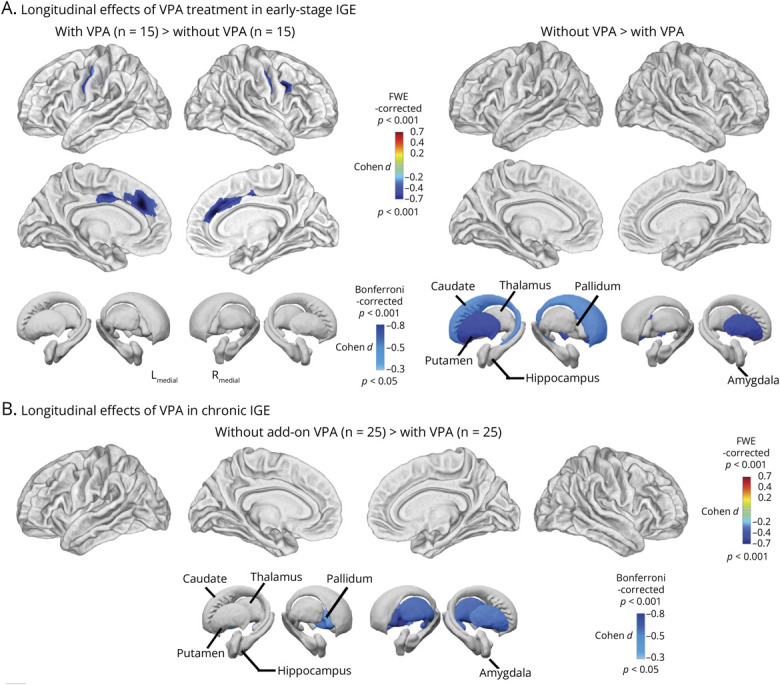
Association With VPA Use (A) In early-stage IGE, participants receiving VPA exhibited progressive cortical thinning in the bilateral precentral gyri compared with those not on VPA (*p* < 0.05, FWE-corrected). By contrast, those not taking VPA showed greater progressive atrophy in the basal ganglia and thalamus (*p* < 0.05, Bonferroni-corrected). (B) In chronic IGE, no significant cortical changes were observed between participants with and without add-on VPA. However, those without add-on VPA showed progressive atrophy in the bilateral pallidum, right putamen, and right thalamus (*p* < 0.05, Bonferroni-corrected). FWE = family-wise error; IGE = idiopathic generalized epilepsy; VPA = valproate.

In the early-stage cohort, 15 participants on VPA were matched to 15 not on VPA. Progressive cortical thinning was observed in the left medial frontal gyrus (1,569 vertices, *p* = 0.004_FWE,_ *d* = −0.68), right inferior frontal lobe (722 vertices, *p* = 0.004_FWE_, *d* = −0.65), bilateral mid-anterior cingulate cortex (left: 1,007 vertices, *p* = 0.010_FWE_, *d* = −0.57; right: 2,665 vertices; *p* = 0.009_FWE_, *d* = −0.65), and precentral gyri (left: 513 vertices, *p* = 0.010_FWE_, *d* = −0.50; right: 825 vertices, *p* = 0.020_FWE_, *d* = −0.56) (Figure 4A, eTables 18 and 19). Subcortically, those not on VPA showed progressive atrophy in the left caudate (*p* = 0.037_Bonferroni_, *d* = −0.45) and the putamen bilaterally (left: *p* < 0.001_Bonferroni_, *d* = −0.75; right: *p* = 0.002_Bonferroni_, *d* = −0.68).

In the chronic cohort, 22 not on VPA showed more subcortical atrophy than those taking VPA (n = 22) between the first and the second scans, including the bilateral pallidum (left: *p* = 0.015_Bonferroni_, *d* = −0.48; right: *p* < 0.001_Bonferroni_, *d* = −0.72), right putamen (*p* = 0.011_Bonferroni_, *d* = −0.55), and right thalamus (*p* = 0.005_Bonferroni_, *d* = −0.60) (Figure 4B, eTable 20).

### Thalamo-Cortical Covariance Analysis

No significant structural covariance findings were observed in the early-stage cohort. In the chronic cohort, the baseline left thalamus exhibited increased covariance with the left postcentral gyrus (511 vertices, *p* = 0.003_FWE_) (Figure 3B). At the repeat scan, the left thalamus correlated with the left superior occipital gyrus (1,786 vertices, *p* < 0.001_FWE_), right superior parietal lobe (614 vertices, *p* = 0.001_FWE_), right precuneus (561 vertices, *p* = 0.001_FWE_), right superior occipital lobe (461 vertices, *p* = 0.001_FWE_), and left precentral gyrus (246 vertices, *p* = 0.009_FWE_) (Figure 3B). Interscan changes in the left thalamus correlated with changes in the left lingual gyrus (214 vertices, *p* = 0.027_FWE_) (Figure 3C). No decreased thalamo-cortical or basal ganglia-cortical correlations were detected.

**Figure 5:**
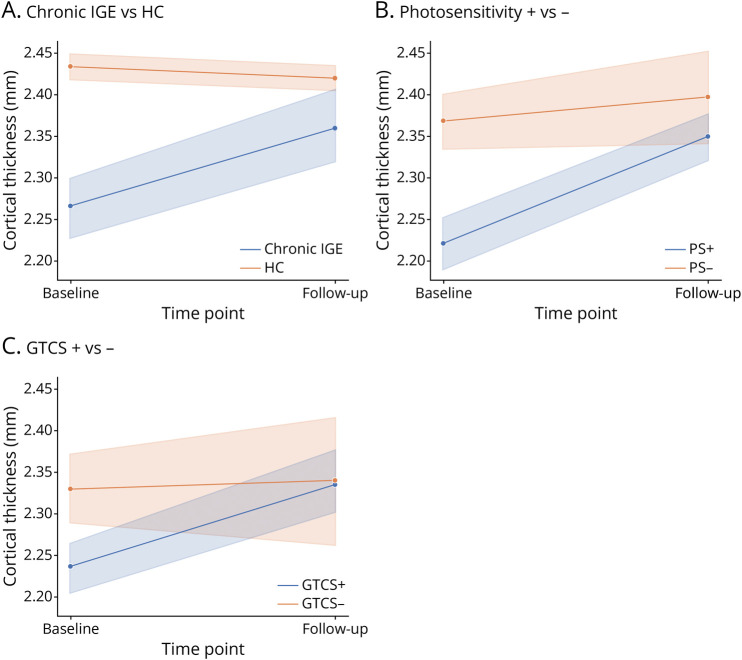
Line Plots of Cortical Thickening in the Right Middle Occipital Gyrus Across Chronic IGE Subgroups (A) Those with chronic showed increased cortical thickness over time compared with controls, who exhibited a slight decrease consistent with age-related thinning. (B) Participants with photosensitivity (PS+) had a more pronounced increase in cortical thickness from baseline to follow-up than those without photosensitivity (PS−). (C) GTCS+ and GTCS− subgroups exhibited cortical thickening over time, with a slightly more significant increase observed in GTCS+ participants. GTCS = generalized tonic-clonic seizure; IGE = idiopathic generalized epilepsy; PS = photosensitivity.

To explore the group-by-time interactions in cortical thickness, we extracted mean cortical thickness values from the significant cluster in the right middle occipital lobe—a region showing longitudinal differences across groups—and examined change trajectories in key clinical subgroups. Figure 5 shows increased thickness over time in chronic IGE, photosensitive, and GTCS groups, contrasting with subtle decline in controls. These diverging trajectories suggest that group effects are driven by differential slopes of change.

## Discussion

We report 2 IGE cohorts—early, largely drug-responsive, and chronic, predominantly uncontrolled—each compared with matched controls. People with chronic IGE showed more extensive progressive cortical and subcortical abnormalities than controls, including motor cortex thickening and thalamic/basal ganglia atrophy associated with ongoing GTCSs, as well as visual cortex thickening linked to photosensitivity.

Our early-stage cohort showed no age-related reduction in cortical thickness, unlike adolescent controls. This accords with a recent longitudinal study in new-onset JME reporting fronto-parietal-temporal increases in cortical thickness, volume, and surface area during the first 2 years after diagnosis.^7^ This pattern likely reflects reduced gray matter pruning and delayed cortical maturation.^26^ The observed pattern may stem from a complex interplay of altered neurodevelopment, seizures, and early ASM exposure.^27^ The observed left-sided effect is cohort-specific, and because all participants were right-handed, handedness could not be modeled.

Although IGE is traditionally viewed as a symmetric disorder, early-stage structural changes may reveal asymmetric vulnerability within cortico-striato-thalamo-cortical networks. Left putaminal atrophy exclusively in right-handed individuals during the early phase of IGE suggests preferential involvement of dominant-hemisphere circuits that support language and executive control. The left putamen forms part of frontal-striatal loops for articulatory planning and cognitive regulation, functions typically left-lateralized in right-handers. This interpretation is supported by fMRI studies in IGE subtypes, which demonstrate aberrant recruitment of motor and premotor networks—including the putamen—during language tasks.^28^ The putamen's role in this network is underscored by its heightened involvement in tonal languages such as Mandarin, where it supports the fine-grained auditory-motor integration essential for speech.^29^ It is also fundamental to second-language acquisition, with its intensive use leading to documented structural adaptations in bilinguals.^30,31^ Taken together, these findings suggest that heightened engagement and early metabolic stress within dominant-hemisphere cortico-striatal circuits may confer selective vulnerability of the left putamen during early IGE, especially in native Mandarin speakers.

In the chronic cohort, frontocentral cortical changes and thalamic/basal ganglia atrophy were linked to ongoing GTCSs, supporting previous cross-sectional findings.^4,12,25^ Unlike the well-controlled early-stage group, participants with chronic IGE showed progressive bilateral thalamic and basal ganglia atrophy, suggesting that these subcortical regions are central to epilepsy-related changes. Structural connectomics studies suggest that subcortical hubs in IGE are particularly vulnerable, contrasting cortical hub vulnerability in TLE.^12^ Thalamo-cortical circuitry contributes to spike-wave and slow-wave discharges.^32^ Atrophy and metabolic dysfunction^33^ in the thalamus and basal ganglia^10,24^ and altered structural connectivity^34^ have been associated with ongoing GTCSs or focal to bilateral tonic-clonic seizures.

We identified visual-cortex thickening in the photosensitive chronic IGE group, consistent with previous cross-sectional findings of photosensitive IGE showing increased occipital thickness^35^ and volumes^36^ compared with nonphotosensitive counterparts. This may reflect pathologic processes such as heightened excitability or impaired inhibition,^37^ or a delayed physiologic thinning.^7^ Imaging studies in IGE showed increased activation and connectivity between motor and occipital areas.^32,38^ This may reflect brain plasticity associated with photosensitivity and GTCSs or adaptive mechanisms to counter excessive cortical excitability.^32^

Thalamic volumes positively correlated with frontal, parietal, and occipital vertices, aligning with previous cross-sectional structural studies.^24^ We observed a progressive increase in structural covariance between the thalamus and visual cortex in IGE with photosensitivity. EEG-fMRI studies show thalamic activity only when photosensitivity is linked to GTCSs.^32^ Gradual visual cortex thickening and frontocentral changes in chronic cases suggest functional overlap between photosensitivity and GTCS circuits.

To explore GTCS and drug resistance, we divided the chronic cohort by the presence of GTCSs between the scans. Those with ongoing GTCSs showed widespread cortical, thalamic, and basal ganglia changes, including precentral thickening and bilateral amygdala atrophy. By contrast, well-controlled patients showed no progressive changes. This confirms that ongoing brain changes are driven by GTCSs rather than disease duration, a finding supported by ENIGMA-Epilepsy studies.^10,39^

Motor region findings have been mixed in IGE, with reports of both thinning and thickening.^24,39,40^ Gray matter loss in motor regions may also relate to VPA effects.^41^ Our AI-driven analysis^5^ suggests a progressive pattern: with ongoing GTCS, atrophy begins in the basal ganglia, then involves the thalamus and cortex, which may predict poor treatment response. The consistent thalamic and basal ganglia atrophy patterns confirm their established role in generalized seizure networks.^42^ The selective vulnerability stems from (1) the thalamus's critical function in generating 3–4 Hz spike-wave oscillations through cortico-thalamo-cortical circuits,^43^ (2) excitotoxic damage from recurrent generalized seizures, and (3) potential neurodevelopmental alterations in GABAergic signaling within basal ganglia-thalamic loops.^44^ The specific involvement of thalamic central nuclei and pallidum aligns with their dense connectivity to cortical areas, generating absence and myoclonic seizures.^24,33^ The topographic specificity suggests that these structural changes may reflect seizure burden and inherent network vulnerability in IGE.^10,45^ This pattern differs from focal epilepsies where atrophy typically localizes to seizure-onset zones, supporting IGE's system-level pathophysiology.^10,39^

In the early-stage VPA group, progressive cortical thinning affected the bilateral precentral gyri, anterior cingulate cortices, and bilateral frontal regions, with no subcortical atrophy. Conversely, those untreated with VPA showed basal ganglia atrophy in the bilateral putamen and left caudate. In the chronic cohort, participants treated with VPA between the scans showed no cortical changes. Similar progressive subcortical patterns were seen in the VPA-treated chronic cohort, with less progressive atrophy in basal ganglia and thalamus regions than those without VPA treatment between the 2 scans. Our previous study indicates that VPA influences cortical morphology in disease-specific areas in IGE,^41^ potentially through selective inhibition of antineoplastic histone deacetylase, which may dysregulate myelination.^46^ Evidence from fMRI^47^ and PET^48^ studies suggests that VPA inhibits seizures by decreasing thalamic and basal ganglia activity. Our longitudinal findings of VPA-related structural changes suggest VPA's “normalizing” role against alterations associated with ongoing GTCSs. This further supports the concept of VPA's network-stabilizing effects, IGE-specific neuroprotection, and superiority in controlling GTCS.^23^ The relatively low VPA utilization in both cohorts (36% early-stage, 38% chronic) may reflect evolving clinical practices, particularly in light of growing awareness of its teratogenic risks and neurodevelopmental concerns.^49^ In the United Kingdom and elsewhere,^49,50^ guidelines increasingly restrict VPA prescribing in women of childbearing potential and younger people, which could explain its modest uptake in the early-stage cohort. For chronic cases, the persistence of suboptimal VPA use might indicate (1) historical prescribing patterns before safety concerns were fully recognized or (2) individualized risk-benefit assessments where alternatives failed. The similar proportions across cohorts suggest a broader transition to newer medications.

We acknowledge the retrospective nature and inherent referral bias in our chronic cohort, as people were referred for chronic and drug-resistant seizures. In addition, syndromes change and progress over time. The difference in intervals between the early and chronic IGE cohorts could have influenced the extent of pathology observed in the chronic group, given that the longer intervals allowed more time for structural changes to manifest. To account for this, we applied statistical adjustments for follow-up duration in our analyses to minimize potential bias. The overlap in basal ganglia alterations between the early and chronic cohorts suggests a shared underlying pathomechanism, with the differences in magnitude likely reflecting varying stages of disease progression. These findings highlight the value of future studies with harmonized follow-up intervals to further clarify the relationship between follow-up duration and disease-specific mechanisms driving structural changes in IGE.

Another limitation of the study is the geographic diversity of the IGE cohorts, with one group from China and the other from England. This introduces some heterogeneity, including geographically matched controls, but it also helps mitigate potential biases and adds a broader context to the findings. We corrected for age and interval differences to minimize the impact of variations in interval differences. The early-stage cohort was not assessed for photosensitivity. Education levels were comparable in controls and the early-stage cohort, but we lacked data on controls' educational attainment or cognitive status for the other control group. An ideal comparator would be a cohort with new-onset IGE in remission after the first ASM.

Third, we examined the effects of uncontrolled GTCSs and photosensitivity but not seizure frequency because of the unreliable seizure counts in IGE. Cognitive and behavioral assessments were not conducted systematically. Future research should test whether progressive cortical and subcortical changes correlate with deficits on serial cognitive assessments.^48^

Our design does not allow for simultaneous evaluation of neurodevelopmental changes in early-stage IGE and cumulative disease effects in chronic IGE; this is compounded by demographic differences, particularly in age and treatment histories. A comprehensive understanding of disease progression would require prospective longitudinal imaging from new-onset adolescence to chronic-stage adulthood, using standardized acquisition protocols—an important future direction our early-stage cohort could inform.

We acknowledge that the case-control contrasts (early IGE vs SLIM; chronic IGE vs PPMI/SPINS) are confounded by site, limiting separation of disease from site effects; these contrasts are, therefore, presented as exploratory, with inference centered on longitudinal within-participant change and within-site subgroup analyses. In this context, including diagnosis as a ComBat covariate is not identifiable and may remove biological variation, so ComBat was used only to mitigate scanner-related variance; this is consistent with the nonsignificant pre/post differences and the unchanged longitudinal effects in sensitivity analyses. We also note limited power in some subgroup analyses (e.g., VPA). Future prospectively balanced, multisite acquisitions will be needed to fully disentangle disease from site effects.

Owing to the study's retrospective nature, we relied on routinely acquired MRI, which did not include diffusion tensor imaging. We, therefore, used structural covariance based on T1-weighted imaging to assess inter-regional connectivity, which has shown good agreement between anatomical connections derived from diffusion tensor imaging tractography and morphometric correlation analysis.^24,25^ This has been applied in IGE previously.^25^

By quantitatively analyzing cortical and subcortical trajectories in a longitudinal design, we identified abnormalities in specific brain regions contributing to chronicity, treatment response, and drug resistance in IGE. Our results suggest the predictive value of measuring cortical thickness and subcortical volumetry as biomarkers for tracking disease progression and ASM therapeutic effects. We identified distinct brain morphology progression between early-stage and chronic IGE. Cortical changes in the frontocentral and temporal regions, alongside progressive atrophy in the thalamus and basal ganglia, are likely associated with chronic drug resistance and active GTCSs. Our study shows cortical thickening in the visual regions of people with chronic IGE, suggesting syndrome-specific characteristics possibly related to the increased prevalence of photosensitivity. Cross-sectional and longitudinal analyses showed VPA-associated thinning in the precentral gyri but less extensive basal ganglia and thalamic atrophy, contrasting our findings in people with ongoing GTCSs. This discrepancy may reflect VPA's advantage over other ASMs in controlling GTCS.^23^ Together, these findings deepen understanding of IGE pathogenesis and inform clinical management strategies. Longitudinal patterns of cortical thinning/thickening that we identified may provide clinically useful markers for IGE management in 2 key aspects: (1) differentiating typical neurodevelopment from disease-associated cortical changes during adolescent/young adult monitoring and (2) identifying candidate neuroanatomical biomarkers (e.g., motor regions) for treatment trials. These findings demonstrate how serial MRI scans can capture dynamic neuroanatomical change that cross-sectional imaging cannot resolve.^19^

## Glossary

ASM: antiseizure medication
CAT12: Computational Anatomy Toolbox
FWE: family-wise error
GTCA: GTCSs-alone
GTCS: generalized tonic-clonic seizure
HC: healthy control
IGE: idiopathic generalized epilepsy
JAE: juvenile absence epilepsy
JME: juvenile myoclonic epilepsy
PPMI: Parkinson's Progression Markers Initiative
SLIM: Southwest University Longitudinal Imaging Multimodal
SPINS: Social Processes Initiative in Neurobiology of the Schizophrenia(s)
VPA: valproate
